# Online version of the self-administered food frequency questionnaire for the Japan Public Health Center-based Prospective Study for the Next Generation (JPHC-NEXT) protocol: Relative validity, usability, and comparison with a printed questionnaire

**DOI:** 10.1016/j.je.2016.08.021

**Published:** 2017-06-16

**Authors:** Erika Kato, Ribeka Takachi, Junko Ishihara, Yuri Ishii, Shizuka Sasazuki, Norie Sawada, Motoki Iwasaki, Yurie Shinozawa, Jun Umezawa, Junta Tanaka, Yuta Yokoyama, Kaori Kitamura, Kazutoshi Nakamura, Shoichiro Tsugane

**Affiliations:** a Department of Community Preventive Medicine, Niigata University Graduate School of Medical and Dental Sciences, Niigata, Japan; b Department of Food Science and Nutrition, Faculty of Human Life and Environment, Nara Women's University, Nara, Japan; c Department of Nutrition Science, Sagami Women's University, Kanagawa, Japan; d Epidemiology and Prevention Group, Center for Public Health Sciences, National Cancer Center, Tokyo, Japan; e Department of Health Promotion Medicine, Niigata University Graduate School of Medical and Dental Sciences, Niigata, Japan

**Keywords:** Usability, Middle-aged adults, Online dietary assessment, Validity, Food frequency questionnaire

## Abstract

**Background:**

Online dietary assessment tools offer advantages over printed questionnaires, such as the automatic and direct data storage of answers, and have the potential to become valuable research methods. We developed an online survey system (web-FFQ) for the existing printed FFQ used in the JPHC-NEXT protocol, the platform of a large-scale genetic cohort study. Here, we examined the validity of ranking individuals according to dietary intake using this web-FFQ and its usability compared with the printed questionnaire (print-FFQ) for combined usage.

**Methods:**

We included 237 men and women aged 40–74 years from five areas specified in the JPHC-NEXT protocol. From 2012 to 2013, participants were asked to provide 12-day weighed food records (12d-WFR) as the reference intake and to respond to the print- and web-FFQs. Spearman's correlation coefficients (CCs) between estimates using the web-FFQ and 12d-WFR were calculated. Cross-classification of intakes was compared with those using the print-FFQ.

**Results:**

Most participants (83%) answered that completing the web-FFQ was comparable to or easier than completing the printed questionnaire. The median value of CCs across energy and 53 nutrients for men and women was 0.47 (range, 0.10–0.86) and 0.46 (range, 0.16–0.69), respectively. CCs for individual nutrient intakes were closely similar to those based on the print-FFQ, irrespective of response location. Cross-classification by quintile of intake based on two FFQs was reasonably accurate for many nutrients and food groups.

**Conclusion:**

This online survey system is a reasonably valid measure for ranking individuals by intake for many nutrients, like the printed FFQ. Mixing of two FFQs for exposure assessments in epidemiological studies appears acceptable.

## Highlights

• We examined validity of intakes using web-FFQ compared with those for the print-FFQ.
• Moderate validities were shown for many estimates based on the web-FFQ.
• These CCs (with 12-day food record) were closely similar to those of the print-FFQ.
• Good concordance between individual rankings were obtained with the two FFQs.
• Mixing of these FFQs for diet survey in epidemiological studies appears acceptable.

## Introduction

Many epidemiological studies that evaluate diet–disease associations, such as large-scale prospective cohort studies, assess the usual long-term diet and rank individuals by intake of specific nutrients using food frequency questionnaires (FFQ).^1^ Typically, a printed questionnaire is sent to the subject and returned to the study office after completion. If many missing responses or logical errors are discovered during study office review, the subject is asked to provide the missing information via telephone or the questionnaire is returned to the subject.^2^ Accepted responses are then converted to electronic data.

With increasing use of the Internet, many dietary assessments, such as FFQs and diet history questionnaires, have been developed using Web technology. Reports on the validity of these assessments have increased dramatically over the last 10 years.^3^, ^4^, ^5^, ^6^, ^7^, ^8^, ^9^, ^10^, ^11^, ^12^, ^13^, ^14^, ^15^, ^16^ However, the subjects in these studies have all been computer-literate young or highly educated individuals, and the use of Web assessment by middle-aged and elderly local populations, which provide the subjects of actual cohort studies, has not been validated.

Web-based dietary assessment tools offer three major advantages. First, the conversion of print questionnaire responses to electronic data is omitted, and data processing is simple and fast.^3^, ^4^, ^5^, ^6^, ^7^ Second, the questionnaire can be sent to many people at once, typically by including a URL for the questionnaire in an e-mail message.^17^ Third, missing responses can be minimized through the use of warnings displayed by a computer program,^3^, ^4^, ^5^, ^6^ obviating the need to check for missing responses or conduct follow-up inquiries and improving data quality and time and cost efficiency. Further, the subject does not need to perform certain tasks, such as crossing out or erasing marked sheet responses, or repeatedly troubleshoot difficulties with the questionnaire with the study administrative office. Even if web-FFQs are restricted to subjects with Internet access,^4^ combined use of web- and print-FFQs in actual cohort populations may help improve response rates and reduce the total burden of large-scale epidemiological studies. To our knowledge, however, the combined use of web- and print-FFQs has not been studied.

We developed an online version of the print questionnaire^18^ used in the baseline survey of the Japan Public Health Center-based Prospective Study for the Next Generation (JPHC-NEXT).^19^ Here, we examined the usability and validity of this on-line questionnaire for a local population within the geographic area specified in the JPHC-NEXT protocol. Further, to examine the mixing of exposure assessments, we compared the estimated intake rankings obtained with the online and print FFQs.

## Methods

### Study settings and participants

The study was conducted in five areas specified in the JPHC-NEXT protocol (Yokote, Saku, Chikusei, Murakami, and Uonuma).^19^ Eligibility criteria were middle-aged and elderly residents of these five areas, as in the JPHC-NEXT protocol. The protocol was approved by the Institutional Review Board of the National Cancer Center, Tokyo, Japan and all other collaborating research institutions.

A total of 255 men and women participated in the study. The 12-day food records and two identical print-FFQs were completed by 253 participants, of whom 250 also completed the web-FFQ. The present validation study was conducted in 237 men and women aged 40–74 years at the start of the study.

### Data collection

To establish a reference intake, participants completed a series of 3-consecutive-day weighed food records, one in each of four seasons (12d-WFR), at intervals of approximately 3 months from November 2012 to December 2013. The self-administered semi-quantitative printed questionnaire (including general information on lifestyle, such as disease history, smoking status, and physical activity, in addition to the FFQ) for the JPHC-NEXT protocol was administered twice between November 2012 and December 2013 at a 1-year interval. The web questionnaire (also including overall information on lifestyle as well as the FFQ) was administered between August and December 2013. Data collection and methods have been described elsewhere.^18^

### Reference method

Each 3-consecutive-day period consisted of 2 weekdays and 1 weekend day. Food portions were measured by each participant during meal preparation using supplied digital scales and measuring spoons and cups. For foods purchased or consumed outside the home, the participants were instructed to record the approximate quantity of all foods in the meal and/or the names of the product and company. To account for the validity of water consumption (from fluids or beverages), water used in soup and in boiled food, as well as drinking water, were also checked. Food records were checked by trained dietitians with the participants on the day after each of the 3-day WFRs on site in each study area and were coded for foods and weights. In some cases, the 3-day WFR was submitted via fax or mail to the study office and checked via telephone.

### Print-FFQ

The print-FFQ consisted of 172 food and beverage items in nine frequency categories and three portion size categories. It asked about the usual consumption of listed foods during the previous year. The food list was initially developed and used for the Japan Public Health Center-based prospective Study^20^, ^21^, ^22^, ^23^, ^24^, ^25^, ^26^ and modified for middle-aged and elderly Japanese residents in a wide variety of areas for use in the JPHC-NEXT Study baseline survey. Details of items and the validity of intake estimates based on the print-FFQ have been described elsewhere.^18^ When staff identified missing answers or errors in the print-FFQ, the participants were asked to provide that information again.

Intakes of energy, 53 nutrients (including water content), and 29 food groups were calculated using the Standard Tables of Food Composition in Japan 2010,^27^ Standard Tables of Food Composition in Japan Fifth Revised and Enlarged Edition 2005 Fatty Acids Section,^28^ and a specifically developed food composition table for isoflavones in Japanese foods.^29^

To compare categories of estimated intake based on the web-FFQ with those based on the print-FFQ, we used data from the second administration, because these FFQs were administered around the same time. To compare usability, the second print questionnaire asked about the total time required to answer the overall information on lifestyle.

### Development and characteristics of the web-FFQ

The web-FFQ is an online self-administered semi-quantitative FFQ. The interface is configured similarly to the print-FFQ, and the structure is the same. With the intention of deriving similar estimates (and validities) from the online version to those from the printed FFQ, we determined not to newly add photographic images of food items or other visual artifices to aid subject recall. Time to complete the questionnaire (including date and time lapsed from when the “start” button was clicked to time the “send data” button was clicked) was measured automatically. The web questionnaire included a question on the ease of use in comparison with the printed questionnaire.

Participants with private or residential internet access (excluding mobile phones) received an e-mail message containing their ID and a unique URL in August 2013. Participants without private or residential internet access completed the web questionnaire via tablet computer or personal computer at a specified site in each area from August to December 2013.

The web questionnaire retained answers entered in preceding pages, allowing completion across different sessions. Programmed alerts were raised if mandatory information was not entered. To check usability, total completion time was compared with the self-reported time required to complete the printed questionnaire.

### Statistical analysis

We included 98 men and 139 women in the main analysis for validity. After exclusion of 27 participants who required 24 h or more to complete the web questionnaire (which allows completion over multiple sessions) or did not provide information on time to complete the printed questionnaire, median values of completion time were compared by sex, age, and response location (private/residential or on-site) among the remaining 86 men and 124 women.

Mean intakes of nutrients and food groups estimated using the web-FFQ were compared to those estimated using the 12d-WFR among the 98 men and 139 women who completed both. To assess agreement in estimated intakes, limits of agreement (LOA) were calculated based-on log-transformed values. The LOA were obtained by overlaying the plot of difference (FFQ − WFR) versus mean ((FFQ + WFR)/2) between the two methods. This was originally termed the Bland–Altman method,^30^ which can also be characterized as the mean difference ±1.96 multiplied by SD of differences. The exponentiated mean difference provided the ratio of intake estimated using the web-FFQ relative to the WFR, with an exponentiated LOA range between 50% and 200% indicating acceptable agreement.^31^ Any dependency between the two methods was tested by fitting the regression line of differences. To determine the validity of the web-FFQ, Spearman's rank correlation coefficients (CCs) between intakes based on the web-FFQ and 12d-WFR were calculated for energy-adjusted values. A residual model was used for energy adjustment.^1^ We corrected the observed CCs for the attenuating effect of random intra-individual error from the usual intake of each energy and nutrient and each food group.^1^, ^32^

Also, to compare categories of estimated intake between the web-FFQ and print-FFQ, we computed the number of participants classified into the same, adjacent, and extreme categories by cross classification according to both quintiles using the web- and print-FFQ.^32^ All analyses were performed using SAS Version 9.4 (SAS Institute Inc., Cary, NC, USA).

## Results

### Study participants

Study participants are characterized in Table 1. Mean age was 57.4 (standard deviation [SD], 8.6) years in men and 56.8 (SD, 8.5) in women. Body mass index was 23.7 (SD, 2.8) kg/m^2^ in men and 22.6 (SD, 3.2) kg/m^2^ in women. Current smokers accounted for 24.5% of men and 2.2% of women. For education and employment, 30.6% of men and 10.1% of women had completed a university degree or higher; 20.4% of men and 31.7% of women worked in professional/technical positions; and 18.4% of men and 18.0% of women worked in clerical positions. Mean time interval was 1.8 (SD, 0.85; median, 2) months in men and 1.6 (SD, 0.83; median, 2) months in women.

**Table 1:** Characteristics of subjects (98 men and 139 women).

	Men	Women
Age, years, mean (SD)	57.4 (8.6)	56.8 (8.5)
Body height, cm, mean (SD)	168.3 (6.9)	156.8 (5.9)^¶¶¶^
Body weight, kg, mean (SD)	67.1 (9.2)	55.6 (8.1)^¶¶¶^
Body mass index, kg/m^2^, mean (SD)	23.7 (2.8)	22.6 (3.2)^¶¶^
Current smoker (%)	24.5	2.2^§§§^
Heavy drinker (%)^a^	34.7	2.9^§§§^
**Education (%)**		^§§§^
Junior high school	6.1	8.6
High school	37.8	35.3
Junior college or vocational school	25.5	46.0
University or higher^b^	30.6	10.1
**Job (%)**		^§§^
Unemployed/homemaker	17.3	31.7
Professional/technical	20.4	31.7
Clerical	18.4	18.0
Sale	1.0	2.1
Service	11.2	6.5
Manufacture	3.1	0.7
Transportation	1.0	0.7
Others^c^	27.6	8.6
**Time required for completion of questionnaires, min**		
Web, median (interquartile range)	63.4 (42.5, 91.4)	81.2 (58.4, 117.8)**
Print, median (interquartile range)	60 (43, 70)	60 (49, 90)*

BMI, body mass index; SD, standard deviation.

^¶¶¶^*p* < 0.001, ^¶¶^*p* < 0.01, tested the difference between sex using the *t*-test; ^§§§^*p* < 0.001, ^§§^*p* < 0.01, tested the difference between sex using the chi-square test; **p < 0.01, *p < 0.05, tested the difference between sex using the Mann–Whitney U test.

a ≥280 g ethanol/wk in men, ≥140 g ethanol/wk in women.

b Including post-graduate degrees.

c Including administrative, agriculture/fishery, and classification impossible.

### Usability of the web-based questionnaire

Of the 253 participants who completed the 12d-WFR and 2 identical print-FFQs, 3 participants did not complete the web-based questionnaire due to technical and network issues. Most participants (83%) answered that the web questionnaire was “very easy (9.3%)”, “easy (53.2%)”, or “almost the same (20.7%)”, compared with the printed questionnaire. Total proportions of answers representing suitable usability of the web-based questionnaire varied by age, with corresponding values of 88%, 86%, 80%, and 72% for those in their 40's, 50's, 60's and 70's, respectively.

Of the 237 participants, 81 without private/residential internet access completed the web questionnaire at the specified site in each area; 30.9% (9 men and 16 women; mean age 67.1; SD, 5.3 years) of these 81 respondents required complete or partial assistance by staff.

Table 2 shows median time to complete the printed and web questionnaires (including overall information on lifestyle) by sex, age, and response location. Participants with private/residential internet access were approximately 7 years younger than those without access. Median time to complete the web questionnaire was similar to that for the printed questionnaire in men, but slightly longer in women, with corresponding values of 63.4 and 60.0 min for men and 81.2 and 60.0 for women, respectively. Although median time to complete the web questionnaire was greater among the respondents on site than for the private/residential respondents in both sexes, the results were similar to those for the printed questionnaire, at 70 and 50 min for men and 90 and 55 min for women, respectively. Median time to complete the web questionnaire among private/residential respondents was closely similar to or slightly longer than that for the printed questionnaire for both sexes.

**Table 2:** Median time to complete the printed questionnaire (self-reported) and web questionnaire (measured automatically, by response location), by sex and age in 86 men and 124 women.^a^

	Men							Women						
	Printed questionnaire		Web questionnaire					Printed questionnaire		Web questionnaire				
			Total	Private/residential (*n* = 58)		On site (*n* = 28)				Total	Private/residential (*n* = 71)		On site (*n* = 53)	
	*n*	min	min	*n*	min	*n*	min	*n*	min	min	*n*	min	*n*	min
40s	17	45.0	46.7	16	44.3	1	96.1	23	45.0	52.4	20	53.8	3	52.4
50s	29	50.0	60.3	21	43.3	8	66.6	50	60.5	72.0	34	68.4	16	101.4
60s	33	70.0	65.3	19	60.4	14	79.5	41	70.0	94.1	16	74.2	25	114.3
70s	7	76.0	88.1	2	128.4	5	72.1	10	110.0	121.9	1	280.3	9	118.8
Median		60.0	63.4		57.4		76.4		60.0	81.2		67.5		111.9

a Remaining after exclusion of 27 participants who required 24 h or more to complete the web questionnaire or who did not provide information on time to complete the printed questionnaire from among the 98 men and 139 women subjects included in the main analysis for validity.

### Estimates of intake by web-FFQ and ranking compared with 12d-WFR

**Fig. 1:**
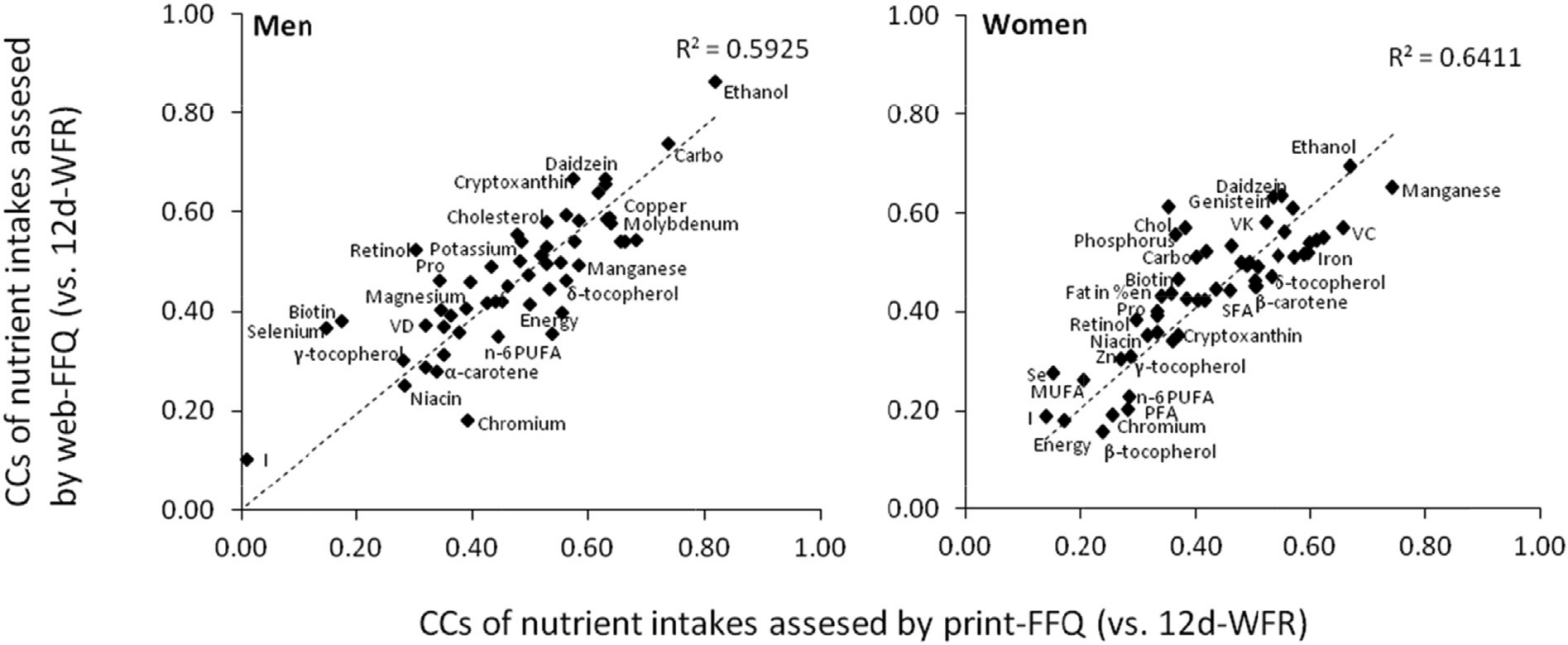
Scatter plot between CCs of the web-FFQ and those of the print-FFQ (vs. 12-day weighed food record for both) for men and women. X-axis: CCs of nutrient intakes (based on energy-adjusted values) assessed using the print-FFQ (vs. 12-day weighed food record); Y-axis: CCs of nutrient intakes (based on energy-adjusted values) assessed using the web-FFQ (vs. 12-day weighed food record). Dotted lines mean regression line. CC, correlation coefficient; FFQ, food frequency questionnaire; WFR, weighed food record.

Table 3, Table 4 show daily intakes of energy and 53 nutrients by the 12d-WFR and web-FFQ, percentage differences between web-FFQ and 12d-WFR, and correlations among men and women. Estimated energy intake levels between the two methods were similar for men (mean percentage: 98%), whereas those based on the web-FFQ was slightly higher among women (112%). Bland–Altman analysis to check agreement of estimated intakes showed that many nutrients were underestimated in men and overestimated in women. Relatively few nutrients and food groups showed an acceptable LOA range between 50% and 200% in their estimates of intake. Regression coefficients were positive for almost all nutrients and statistically significant for both men and women. This indicates that agreement in the estimation of intake became worse with increasing intake. The deattenuated CC of total energy intake in women was lower than in men. The CCs of deattenuated energy-adjusted values varied from 0.10 for iodine to 0.86 for ethanol in men, and from 0.16 for beta-tocopherol to 0.69 for ethanol in women. Median CC across energy and the 53 nutrients was 0.47 in men and 0.46 in women. These CCs for energy and the individual nutrients between intakes from the web-FFQ and 12d-WFR were closely similar to those between the print-FFQ and 12d-WFR,^18^ with corresponding median CCs of 0.50 and 0.43, respectively (data not shown). Pearson's correlation coefficient between these CCs was 0.81 for men and 0.84 for women (Fig. 1).

**Table 3:** Comparison of nutrient intakes using the web-based food frequency questionnaire (web-FFQ) and 12-day WFR based on agreement, ranking correlations, and joint classification by quintile in men (*n* = 98).

	12-day WFR			Web-FFQ			Bland–Altman method: difference in %^a^					Spearman rank CC^d^^,^^e^^,^^f^	Joint classification by Q5^g^		
	Mean	SD	Median	Mean	SD	Median	Mean	95% CI	LOA^b^		Slope^c^		Same	Same & adjacent	Extreme
Energy, kcal	2315	(447)	2244	2358	(928)	2234	98	92, 104	54	178	0.71**	0.42**	33	62	0
Water content, g	2683	(644)	2663	2679	(909)	2533	97	90, 104	48	195	0.50**	0.29**	31	61	3
Protein, g	83.7	(18.1)	82.9	79.3	(38.6)	69.8	89	83, 96	44	182	0.80**	0.46**	33	69	2
The sum of amino acid residues, g	29.4	(7.1)	29.6	29.3	(9.3)	29.0	98	92, 104	54	177	0.31*	0.39**	28	63	2
Total fat, g	62.6	(16.9)	59.6	70.2	(62.7)	56.8	98	88, 109	35	272	0.96**	0.50**	31	74	4
Total fat in % energy	24.2	(3.9)	24.4	25.1	(8.2)	24.5	100	94, 106	56	177	0.86**	0.45**	32	70	2
Saturated fatty acid, g	17.1	(5.4)	16.9	21.0	(20.3)	16.0	105	94, 118	36	312	0.88**	0.54**	31	69	3
Monounsaturated fatty acid, g	22.8	(6.5)	22.1	26.7	(26.2)	20.3	100	90, 112	34	295	0.99**	0.50**	27	78	2
Polyunsaturated fatty acid, g	13.6	(3.6)	13.1	14.4	(10.4)	12.2	97	89, 106	41	229	0.87**	0.42**	23	67	2
n-3 PUFA, g	2.9	(0.9)	2.8	2.6	(1.2)	2.4	89	83, 95	45	177	0.45**	0.36**	27	64	2
n-6 PUFA, g	10.5	(2.9)	9.8	11.8	(9.6)	9.9	102	93, 111	42	245	0.84**	0.35**	28	61	1
Triacylglycerol equivalents, g	54.8	(15.0)	52.6	64.8	(58.9)	52.4	103	93, 114	36	291	0.96**	0.47**	32	73	5
Cholesterol, mg	369.4	(117)	362.8	341.7	(345)	262.2	78	69, 87	26	229	0.81**	0.58**	35	74	1
Carbohydrate, g	300.1	(63.1)	291.7	283.7	(83.6)	280.7	92	88, 97	55	153	0.49**	0.74**	41	78	1
Total dietary fiber, g	16.8	(5.7)	16.0	12.6	(6.1)	11.4	73	68, 79	34	158	0.48**	0.54**	40	74	3
Water soluble fiber, g	3.7	(1.3)	3.6	3.0	(1.6)	2.6	81	76, 86	43	153	0.53**	0.58**	50	77	2
Water insoluble fiber, g	12.4	(4.3)	11.9	9.2	(4.3)	8.7	74	68, 79	36	152	0.37**	0.54**	36	78	4
Sodium, mg	4570	(1092)	4437	4305	(2291)	3871	87	79, 94	37	201	0.88**	0.41**	27	65	2
Potassium, mg	3105	(887)	3085	2894	(1206)	2522	90	84, 97	44	184	0.41**	0.50**	32	70	4
Calcium, mg	570	(182)	571	552	(370)	476	88	80, 97	35	221	0.60**	0.54**	32	73	1
Magnesium, mg	325	(86)	321	324	(116)	303	97	91, 104	52	181	0.39**	0.41**	35	70	3
Phosphorus, mg	1258	(290)	1235	1221	(543)	1105	92	86, 99	47	182	0.66**	0.56**	34	73	1
Iron, mg	9.5	(2.5)	9.6	9.0	(3.5)	8.2	92	87, 98	51	167	0.44**	0.53**	37	72	3
Zinc, mg	9.6	(2.3)	9.3	9.5	(4.8)	8.2	94	88, 101	48	185	0.75**	0.42**	29	71	4
Copper, mg	1.44	(0.36)	1.38	1.28	(0.42)	1.21	93	90, 96	68	128	0.29*	0.59**	37	80	1
Manganese, mg	4.53	(1.55)	4.23	4.00	(1.60)	3.64	89	84, 95	50	160	0.24	0.49**	39	74	2
Iodine, μg	1934	(3976)	517	220	(279)	155	22	15, 32	1	787	−0.69**	0.10	21	56	7
Selenium, μg	61	(19)	59	66	(32)	59	103	94, 113	42	252	0.43**	0.37**	28	66	4
Chromium, μg	8	(3)	7	6	(3)	6	84	76, 91	35	201	0.41*	0.18	27	64	6
Molybdenum, μg	216	(68)	196	234	(81)	220	106	99, 113	56	198	0.32**	0.58**	35	72	1
Retinol, μg	267	(346)	161	377	(448)	174	120	99, 145	19	768	0.37**	0.52**	31	71	2
α-carotene, μg	498	(295)	412	374	(293)	280	64	52, 79	8	505	0.60**	0.28**	24	62	2
β-carotene, μg	3649	(1703)	3289	2493	(1741)	1936	61	53, 70	16	229	0.50**	0.40**	32	67	3
Cryptoxanthin, μg	315	(348)	216	526	(635)	322	134	105, 171	13	1406	0.33**	0.67**	33	73	0
β-carotene equivalents, μg	4263	(1975)	3974	2951	(2009)	2247	62	55, 71	17	225	0.45**	0.45**	29	69	3
Retinol equivalents, μg	639	(380)	565	626	(491)	500	86	75, 98	22	330	0.51**	0.37**	36	61	3
Vitamin D, μg	11.31	(5.21)	10.59	8.91	(5.60)	7.64	76	68, 85	25	234	0.38**	0.37**	27	64	4
α-tocopherol, mg	8.5	(2.6)	8.4	7.7	(4.3)	6.7	86	80, 93	40	187	0.63**	0.51**	34	73	2
β-tocopherol, mg	0.4	(0.1)	0.4	0.4	(0.2)	0.4	101	99, 104	78	130	0.59**	0.36**	27	60	1
γ-tocopherol, mg	11.1	(3.3)	10.1	11.6	(8.5)	10.0	95	87, 105	38	240	0.81**	0.30**	31	60	2
δ-tocopherol, mg	2.9	(1.0)	2.6	2.6	(1.4)	2.3	89	83, 95	45	175	0.56**	0.46**	30	68	1
Vitamin K, μg	298	(132)	280	254	(148)	213	79	71, 87	28	218	0.42**	0.59**	38	73	2
Vitamin B_1_, mg	1.26	(0.52)	1.12	1.04	(0.52)	0.93	90	85, 95	54	150	0.18	0.31**	29	67	4
Vitamin B_2_, mg	1.68	(0.62)	1.61	1.43	(0.77)	1.30	89	85, 95	52	154	0.36*	0.49**	30	69	1
Niacin, mg	23.7	(6.9)	23.0	24.7	(12.0)	21.9	100	92, 108	47	211	0.49**	0.25*	24	54	3
Vitamin B_6_, mg	1.84	(0.91)	1.66	1.61	(0.69)	1.50	93	88, 98	53	161	−0.15	0.42**	32	66	3
Vitamin B_12_, μg	9.8	(4.3)	9.4	8.3	(5.0)	7.4	81	73, 90	29	230	0.35**	0.46**	34	65	1
Folate, μg	453	(159)	433	356	(160)	312	76	70, 82	36	162	0.32**	0.59**	37	72	1
Pantothenic acid, mg	7.16	(1.84)	6.91	7.59	(3.55)	7.03	101	95, 108	55	186	0.60**	0.64**	34	83	0
Biotin, μg	34.95	(10.3)	35.38	40.46	(15.0)	37.30	113	106, 121	58	219	0.24	0.38**	32	62	3
Vitamin C, mg	142	(71)	126	105	(71)	88	68	61, 77	22	212	0.38**	0.54**	33	72	3
Daidzein, mg	13.92	(8.51)	11.41	15.12	(9.98)	11.68	101	91, 112	36	281	0.31**	0.67**	31	73	0
Genistein, mg	23.4	(14.3)	19.4	24.8	(16.8)	18.6	97	87, 109	33	287	0.34**	0.66**	36	71	0
Ethanol, g	27.4	(24.9)	20.7	32.7	(30.0)	27.0	114	98, 132	26	504	0.05	0.86**	46	93	0
Median												0.47	32	70	2

12d-WFR, 12-day weighed food records; SD, standard deviation.

** Expressed as *p* values of <0.01, * as *p* values of <0.05.

a Exponential transform [mean(Web-FFQ − 12d-WFR)] as a multiple of the WFR (all dietary intake data were log-transformed).

b Mean difference ±1.96*(standard deviation of differences).

c Regression coefficient of the mean of two methods regressed on the difference between the methods.

d Spearman's rank correlation coefficients based on energy-adjusted values (other than energy intake and total fat in %energy) and expressed as deattenuated CC.

e Deattenuated CCx = observed CCx*SQRT(1 + *λx*/*n*), where *λx* is the ratio of within-to between-individual variance for nutrient *x*, and *n* is number of food records.

f *p* values were for Spearman's CCs of energy-adjusted intake.

g Joint classification by quintile, expressed as a percentage.

**Table 4:** Comparison of energy and nutrient intakes using the web-based food frequency questionnaire (web-FFQ) and 12d-WFR based on agreement, ranking correlations, and joint classification by quintile in women (*n* = 139).

	12d-WFR			Web-FFQ			Bland–Altman method: difference in %^a^					Spearman rank CC^d^^,^^e^^,^^f^	Joint classification by Q5^g^		
	Mean	SD	Median	Mean	SD	Median	Mean	95% CI	LOA^b^		Slope^c^		Same	Same & adjacent	Extreme
Energy, kcal	1807	(307)	1746	2077	(763)	1964	110	103, 116	55	220	1.13**	0.18*	27	55	6
Water content, g	2324	(548)	2272	2696	(1157)	2478	109	102, 117	50	239	0.78**	0.50**	33	71	1
Protein, g	70.0	(14.6)	68.6	79.3	(37.2)	67.4	105	98, 113	46	242	1.11**	0.40**	29	65	4
The sum of amino acid residues, g	24.0	(5.7)	23.8	31.3	(13.9)	28.5	122	115, 131	57	264	0.80**	0.47**	27	67	3
Total fat, g	54.6	(14.0)	51.0	69.7	(40.5)	61.0	114	105, 125	41	315	1.11**	0.39**	30	64	4
Total fat in % energy	27.0	(4.0)	26.7	28.7	(6.8)	27.8	104	100, 108	66	164	0.74**	0.43**	32	65	3
Saturated fatty acid, g	15.2	(4.9)	14.4	21.7	(17.4)	17.1	121	111, 133	42	348	1.01**	0.44**	27	69	4
Monounsaturated fatty acid, g	19.2	(5.1)	18.4	25.4	(14.0)	21.8	119	109, 130	43	328	1.06**	0.26**	27	64	6
Polyunsaturated fatty acid, g	11.7	(2.9)	11.1	14.3	(6.3)	13.3	114	105, 122	47	274	1.02**	0.20*	26	60	6
n-3 PUFA, g	2.4	(0.8)	2.3	2.7	(1.3)	2.4	107	101, 114	53	218	0.69**	0.42**	26	68	4
n-6 PUFA, g	9.2	(2.4)	8.6	11.5	(5.1)	10.6	116	108, 125	49	276	1.00**	0.23*	26	56	4
Triacylglycerol equivalents, g	47.1	(12.2)	44.0	64.3	(37.6)	56.2	122	112, 133	44	337	1.09**	0.36**	27	65	4
Cholesterol, mg	304.3	(88.7)	288.0	312.0	(208)	265.2	89	80, 99	27	299	1.05**	0.57**	33	72	1
Carbohydrate, g	248.5	(40.5)	243.8	270.3	(78.7)	260.2	105	100, 111	58	191	0.94**	0.51**	37	71	2
Total dietary fiber, g	16.5	(5.1)	15.6	16.9	(7.8)	15.5	96	89, 103	41	223	0.81**	0.54**	32	73	2
Water soluble fiber, g	3.7	(1.4)	3.6	4.0	(2.0)	3.6	101	95, 108	48	212	0.79**	0.52**	31	71	2
Water insoluble fiber, g	12.1	(3.7)	11.7	12.2	(5.5)	11.4	95	89, 102	43	210	0.75**	0.54**	32	72	1
Sodium, mg	3801	(922)	3697	4489	(1980)	4231	109	100, 118	40	294	1.07**	0.43**	32	69	3
Potassium, mg	2963	(786)	2917	3630	(1754)	3284	112	104, 121	47	271	0.86**	0.51**	37	70	1
Calcium, mg	588	(203)	575	828	(784)	616	114	103, 126	35	371	0.99**	0.52**	31	71	1
Magnesium, mg	294	(77)	290	354	(145)	336	114	107, 122	53	246	0.75**	0.49**	37	68	2
Phosphorus, mg	1091	(245)	1053	1340	(777)	1140	111	103, 120	46	268	1.07**	0.55**	40	78	1
Iron, mg	8.8	(2.5)	8.7	9.4	(3.4)	8.7	103	97, 109	53	199	0.63**	0.51**	39	73	2
Zinc, mg	7.9	(1.6)	7.8	9.2	(4.1)	8.2	108	102, 115	55	214	1.01**	0.30**	28	63	4
Copper, mg	1.25	(0.29)	1.22	1.36	(0.46)	1.30	104	101, 107	72	148	0.63**	0.50**	32	68	2
Manganese, mg	4.17	(1.46)	3.82	4.08	(1.58)	3.82	97	93, 102	57	164	0.21*	0.65**	42	76	1
Iodine, μg	1663	(3440)	549	287	(293)	183	33	25, 45	1	1000	−0.67**	0.19	27	63	7
Selenium, μg	48	(12)	47	64	(34)	55	122	112, 134	43	345	1.08**	0.27*	27	54	4
Chromium, μg	7	(2)	7	7	(4)	7	101	93, 109	41	250	0.91**	0.19	23	59	7
Molybdenum, μg	171	(52)	162	227	(109)	202	127	119, 135	62	260	0.46**	0.61**	38	75	1
Retinol, μg	201	(176)	148	328	(407)	182	128	111, 147	25	662	0.62**	0.38**	29	68	6
α-carotene, μg	439	(256)	368	728	(1111)	524	111	94, 131	15	797	0.86**	0.56**	32	68	3
β-carotene, μg	3656	(1535)	3551	4406	(3962)	3503	98	87, 111	23	417	0.78**	0.46**	32	69	1
Cryptoxanthin, μg	446	(349)	385	1138	(1250)	731	212	170, 264	16	2734	0.38**	0.35**	26	63	3
β-carotene equivalents, μg	4270	(1748)	4202	5341	(4665)	4548	103	91, 116	24	433	0.81**	0.45**	29	69	1
Retinol equivalents, μg	573	(256)	521	776	(607)	616	114	102, 128	31	427	0.78**	0.42**	30	66	1
Vitamin D, μg	8.89	(4.94)	8.27	9.58	(6.98)	7.69	99	89, 110	28	349	0.42**	0.49**	29	73	2
α-tocopherol, mg	8.0	(2.6)	7.7	9.1	(4.4)	8.6	105	98, 113	44	254	0.85**	0.45**	35	66	4
β-tocopherol, mg	0.3	(0.1)	0.3	0.4	(0.2)	0.4	106	103, 108	81	138	1.09**	0.16	22	53	4
γ-tocopherol, mg	10.1	(2.9)	9.5	12.4	(5.9)	10.9	112	104, 122	43	291	0.95**	0.31**	27	62	4
δ-tocopherol, mg	2.6	(0.9)	2.5	2.9	(1.7)	2.5	102	96, 109	50	210	0.73**	0.47**	31	65	4
Vitamin K, μg	293	(111)	276	341	(237)	296	101	92, 112	32	322	0.75**	0.58**	33	70	1
Vitamin B_1_, mg	1.02	(0.36)	0.96	1.11	(0.49)	1.06	103	99, 107	66	162	0.61**	0.34**	27	65	3
Vitamin B_2_, mg	1.50	(0.44)	1.41	1.75	(1.18)	1.47	104	99, 110	56	194	1.01**	0.45**	31	66	3
Niacin, mg	18.4	(5.3)	17.9	22.6	(9.2)	21.2	116	109, 125	52	260	0.69**	0.35**	27	64	3
Vitamin B_6_, mg	1.45	(0.58)	1.35	1.63	(0.65)	1.58	106	102, 111	66	170	0.38**	0.52**	27	68	1
Vitamin B_12_, μg	7.3	(3.4)	6.9	7.9	(6.2)	6.8	99	90, 108	34	288	0.52**	0.61**	35	70	0
Folate, μg	444	(147)	425	457	(216)	422	96	89, 103	40	231	0.69**	0.55**	29	69	1
Pantothenic acid, mg	6.36	(1.55)	6.25	8.49	(4.55)	7.33	120	112, 128	56	255	1.04**	0.53**	35	73	2
Biotin, μg	31.36	(8.51)	30.07	43.44	(21.4)	41.04	128	119, 137	57	288	0.75**	0.44**	28	66	3
Vitamin C, mg	155	(73)	145	171	(103)	153	99	90, 109	33	302	0.66**	0.57**	33	76	2
Daidzein, mg	13.19	(7.30)	11.52	17.74	(14.9)	13.99	119	108, 131	39	365	0.41**	0.63**	35	72	3
Genistein, mg	22.4	(12.5)	19.7	29.3	(25.1)	22.4	115	104, 127	35	378	0.42**	0.63**	37	73	3
Ethanol, g	4.7	(9.7)	0.9	5.2	(10.2)	0.0	91	82, 102	27	315	0.15**	0.69**	44	83	0
Median												0.46	31	68	3

12d-WFR, 12-day weighed food records; SD, standard deviation.

** Expressed as *p* values of <0.01, * as *p* values of <0.05.

a Exponential transform [mean(Web-FFQ − 12d-WFR)] as a multiple of the WFR (all dietary intake data were log-transformed).

b Mean difference ±1.96*(standard deviation of differences).

c Regression coefficient of the mean of two methods regressed on the difference between the methods.

d Spearman's rank correlation coefficients based on energy-adjusted values (other than energy intake and total fat in %energy) and expressed as deattenuated CC.

e Deattenuated CCx = observed CCx*SQRT(1 + *λx*/*n*), where *λx* is the ratio of within-to between-individual variance for nutrient *x*, and *n* is number of food records.

f *p* values were for Spearman's CCs of energy-adjusted intake.

g Joint classification by quintile, expressed as a percentage.

Table 5, Table 6 also show these results for 29 food groups. With regard to agreement in estimating food group intakes, many items were either under- or overestimated in both men and women. Also, positive regression coefficients were statistically significant for many food groups in both men and women. The CCs of deattenuated energy-adjusted values varied from 0.09 for algae to 0.74 for alcoholic beverages in men and from 0.07 for fats and oils to 0.77 for green tea in women. Median CC across 29 food groups was 0.48 for men and 0.44 for women. On cross classification by quintile, however, almost all nutrients and food groups were classified into their respective opposite extreme category by 5% or lower in men or women, with corresponding median values of 2% and 3% for nutrients, and 3% and 3% for food groups, respectively.

**Table 5:** Comparison of food group intakes using the web-based food frequency questionnaire (web-FFQ) and 12d-WFR based on agreement, ranking correlations, and joint classification by quintile in men (*n* = 98).

	12d-WFR				Web-FFQ				Bland–Altman method: difference in %^a^					Spearman rank CC^d^^,^^e^^,^^f^	Joint classification by Q5^g^		
	Mean	SD	Median		Mean	SD	Median		Mean	95% CI	LOA^b^		Slope^c^		Same	Same & adjacent	Extreme
**Men (*n* = 98)**																	
Cereals	508	(138)		495	588	(213)		588	112	105, 119	61	203	0.43**	0.70**	40	77	0
Rice	394	(144)		380	430	(184)		491	105	97, 113	51	217	0.29**	0.66**	36	76	1
Potatoes and starches	45	(29)		40	28	(21)		24	56	46, 69	8	392	0.49**	0.32*	24	64	5
Sugar	6	(3)		5	0	(2)		0	21	18, 23	6	68	−0.17	0.27*	30	64	3
Pulses	70	(47)		56	66	(62)		43	78	66, 91	16	368	0.54**	0.59**	30	76	1
Vegetables	358	(163)		331	236	(177)		194	55	48, 63	15	204	0.74**	0.55**	33	73	2
Green & yellow	123	(67)		109	114	(110)		73	71	60, 85	13	391	0.66**	0.49**	32	73	3
White	234	(119)		222	123	(98)		99	45	39, 52	12	174	0.72**	0.44**	32	70	3
Pickled	14	(13)		10	23	(26)		16	142	112, 180	14	1460	0.37*	0.41**	32	70	4
Cruciferous	118	(71)		103	45	(34)		33	34	29, 40	7	172	0.53**	0.31**	26	61	4
Fruits	94	(79)		71	126	(128)		86	124	99, 156	14	1133	0.06	0.58**	36	78	1
Citrus fruit	22	(30)		11	41	(52)		23	170	124, 233	8	3676	0.17	0.54**	31	71	1
Other fruit	72	(66)		53	83	(95)		50	112	86, 144	9	1359	−0.08	0.61**	37	76	1
Fungi	21	(16)		18	9	(9)		6	40	31, 51	4	431	0.27	0.16	19	55	6
Algae	10	(11)		8	7	(8)		6	70	56, 88	8	646	0.26	0.09	21	52	5
Fish and shellfish	108	(44)		107	74	(49)		61	61	53, 70	15	242	0.58**	0.41**	34	69	5
Meats	87	(36)		84	107	(161)		65	90	76, 106	18	452	0.88**	0.47**	26	65	2
Processed meat	17	(12)		16	11	(15)		8	55	46, 67	8	363	0.32*	0.39**	24	62	3
Red meat	46	(23)		44	65	(109)		38	95	78, 115	14	628	0.81**	0.58**	26	66	0
Poultry	21	(16)		18	29	(54)		15	106	81, 140	7	1542	0.26	0.17	19	58	6
Eggs	40	(18)		39	37	(60)		25	67	57, 79	14	320	0.78**	0.54**	29	71	3
Milk and dairy products	105	(84)		87	216	(300)		139	170	136, 213	19	1532	0.17	0.68**	37	80	0
Fats and oils	12	(5)		11	13	(11)		11	102	90, 116	30	345	0.37*	0.31**	24	64	3
Confectionaries	31	(27)		22	16	(17)		11	49	38, 63	4	597	0.06	0.55**	29	72	2
Alcoholic beverages	350	(313)		302	414	(342)		382	109	83, 142	8	1521	0.11	0.74**	47	83	0
Non-alcoholic beverages	600	(385)		543	588	(363)		480	111	90, 138	14	901	−0.76**	0.17	27	56	6
Green tea	314	(335)		220	312	(361)		174	86	58, 127	2	3872	0.14	0.56**	42	74	2
Coffee	123	(148)		69	217	(184)		174	261	185, 370	9	7751	−0.10	0.48**	32	68	2
Seasonings and spices	138	(74)		124	22	(12)		19	16	14, 18	5	52	0.13	0.36**	26	66	5
Median														0.48	30	70	3

12d-WFR, 12-day weighed food records; SD, standard deviation.

** Expressed as *p* values of <0.01, * as *p* values of <0.05.

a Exponential transform [mean(Web-FFQ − 12d-WFR)] as a multiple of the WFR (all dietary intake data were log-transformed).

b Mean difference ±1.96*(standard deviation of differences).

c Regression coefficient of the mean of two methods regressed on the difference between the methods.

d Spearman's rank correlation coefficients based on energy-adjusted values and expressed as deattenuated CC.

e Deattenuated CCx = observed CCx*SQRT(1 + *λx*/*n*), where *λx* is the ratio of within-to between-individual variance for nutrient *x*, and *n* is number of food records.

f *p* values were for CCs of energy-adjusted intake.

g Joint classification by quintile, expressed as a percentage.

**Table 6:** Comparison of food group intakes using the web-based food frequency questionnaire (web-FFQ) and 12d-WFR based on agreement, ranking correlations, and joint classification by quintile in women (*n* = 139).

	12d-WFR				Web-FFQ				Bland–Altman method: difference in %^a^					Spearman Rank CC^d^^,^^e^^,^^f^	Joint classification by Q5^g^		
	Mean	SD	Median		Mean	SD	Median		Mean	95% CI	LOA^b^		Slope^c^		Same	Same & adjacent	Extreme
**Women (*n* = 139)**																	
Cereals	349	(81)		495	450	(120)		588	127	121, 134	69	236	0.26*	0.37**	29	62	3
Rice	259	(86)		380	319	(110)		491	120	112, 130	50	288	0.20*	0.55**	35	75	4
Potatoes and starches	43	(25)		40	43	(46)		24	79	68, 92	14	466	0.79**	0.44**	29	71	5
Sugar	7	(3)		5	0	(1)		0	19	17, 20	7	52	−0.38**	0.38**	31	64	4
Pulses	70	(43)		56	78	(71)		43	93	83, 106	22	391	0.49**	0.64**	40	72	1
Vegetables	342	(131)		331	351	(213)		194	89	80, 99	26	312	0.81**	0.42**	24	65	3
Green & yellow	125	(61)		109	160	(113)		73	107	94, 122	23	487	0.69**	0.45**	30	65	2
White	217	(95)		222	191	(130)		99	76	67, 85	19	300	0.83**	0.33**	28	67	5
Pickled	14	(16)		10	35	(40)		16	184	150, 226	17	1981	0.49**	0.47**	29	66	2
Cruciferous	118	(64)		103	71	(63)		33	47	40, 56	6	340	1.03**	0.36**	27	62	3
Fruits	138	(85)		71	233	(186)		86	145	123, 171	22	973	0.36**	0.50**	32	70	2
Citrus fruit	36	(32)		11	89	(96)		23	219	171, 279	13	3805	0.06	0.32**	24	60	4
Other fruit	102	(69)		53	143	(124)		50	123	105, 145	19	815	0.29**	0.54**	32	71	0
Fungi	18	(12)		18	16	(15)		6	80	69, 93	13	485	0.16	0.41**	29	63	2
Algae	9	(10)		8	9	(10)		6	102	85, 122	12	833	0.03	0.22*	30	57	5
Fish and shellfish	81	(35)		107	74	(52)		61	78	69, 89	17	357	0.71**	0.59**	31	72	1
Meats	62	(27)		84	71	(55)		65	95	82, 109	19	484	0.64**	0.28**	27	63	6
Processed meat	12	(8)		16	10	(9)		8	75	65, 86	15	379	0.24**	0.62**	31	72	1
Red meat	32	(18)		44	39	(37)		38	95	80, 113	13	706	0.71**	0.31**	24	59	4
Poultry	17	(11)		18	22	(21)		15	107	89, 129	12	956	0.26*	0.49**	23	64	5
Eggs	32	(14)		39	30	(32)		25	75	65, 86	15	383	0.93**	0.60**	32	68	1
Milk and dairy products	147	(101)		87	388	(603)		139	167	142, 197	25	1127	0.50**	0.60**	39	77	3
Fats and oils	10	(4)		11	14	(7)		11	130	117, 145	37	465	0.73**	0.07	25	58	8
Confectionaries	45	(32)		22	28	(23)		11	50	41, 61	5	509	0.83**	0.36**	27	63	3
Alcoholic beverages	83	(167)		302	104	(218)		382	60	46, 78	3	1423	0.21**	0.66**	38	77	0
Non-alcoholic beverages	678	(383)		543	667	(551)		480	93	82, 105	21	407	0.21	0.43**	32	67	3
Green tea	393	(333)		220	365	(361)		174	83	67, 104	7	1048	0.14	0.77**	53	86	1
Coffee	121	(179)		69	229	(294)		174	243	185, 320	10	6072	0.00	0.61**	32	71	1
Seasonings and spices	112	(71)		124	23	(12)		19	22	19, 25	6	86	0.05	0.28**	33	60	3
Median														0.44	30	66	3

12d-WFR, 12-day weighed food records; SD, standard deviation.

** Expressed as *p* values of <0.01, * as *p* values of <0.05.

a Exponential transform [mean(Web-FFQ − 12d-WFR)] as a multiple of the WFR (all dietary intake data were log-transformed).

b Mean difference ±1.96*(standard deviation of differences).

c Regression coefficient of the mean of two methods regressed on the difference between the methods.

d Spearman's rank correlation coefficients based on energy-adjusted values and expressed as deattenuated CC.

e Deattenuated CCx = observed CCx*SQRT(1 + *λx*/*n*), where *λx* is the ratio of within-to between-individual variance for nutrient *x*, and *n* is number of food records.

f *p* values were for CCs of energy-adjusted intake.

g Joint classification by quintile, expressed as a percentage.

### Cross-classification by quintile compared with print-FFQ

We further compared agreement of the categorization of estimated intake by the two different FFQs administered at an average interval of 1.7 (SD, 0.8) months based on cross-classification by quintile (Table 7, Table 8). Nutrients and food groups were classified into their opposite extreme categories by 5% or less of men or women, with corresponding median values for men and women of 1% and 2% for nutrients, and 1% and 1% for food groups, respectively. In addition, classification into the same and adjacent categories for nutrients ranged from 57% for total fat in percentage of energy derived from fats to 97% for ethanol in men and from 64% for selenium to 93% for ethanol in women; for food groups, classification into the same and adjacent categories ranged from 66% for fats and oils to 91% for alcoholic beverages in men and from 60% for red meat to 91% for coffee in women. Median values of the same and adjacent categories for nutrients were 77% in men and 75% in women; corresponding values for food groups were and 74% in men and 75% in women.

**Table 7:** Comparison of the web-FFQ and print-FFQ for energy-adjusted intake of nutrients, based on correlation coefficient and cross-classification by quintile (%).

	Men (*n* = 98)				Women (*n* = 139)			
	CCs^a^	Same category	Same and adjacent category	Extreme category	CCs^a^	Same category	Same and adjacent category	Extreme category
Energy^b^	0.58	34	71	0	0.59	37	81	1
Water content	0.43	33	70	1	0.64	42	79	1
Protein	0.53	46	72	2	0.47	33	78	4
The sum of amino acid residues	0.58	31	80	0	0.56	33	75	3
Total fat	0.49	33	73	1	0.55	40	75	1
Total fat in % energy^b^	0.50	23	57	5	0.59	29	66	2
Saturated fatty acid	0.49	34	67	2	0.56	39	78	2
Monounsaturated fatty acid	0.50	34	66	0	0.46	39	69	1
Polyunsaturated fatty acid	0.46	36	63	1	0.42	36	67	3
n-3 PUFA	0.50	27	72	0	0.49	36	74	2
n-6 PUFA	0.45	35	65	0	0.42	33	69	4
Triacylglycerol equivalents	0.49	32	72	1	0.54	38	74	2
Cholesterol	0.61	36	80	1	0.51	35	74	2
Carbohydrate	0.60	44	77	1	0.61	36	81	1
Total dietary fiber	0.58	46	77	2	0.61	40	75	1
Water soluble fiber	0.56	50	77	1	0.59	34	73	1
Water insoluble fiber	0.58	41	77	2	0.61	43	73	1
Sodium	0.58	39	78	1	0.60	37	78	1
Potassium	0.68	43	82	0	0.61	34	79	2
Calcium	0.61	38	78	0	0.49	34	73	3
Magnesium	0.67	41	80	1	0.63	37	80	1
Phosphorus	0.61	35	72	0	0.47	30	72	1
Iron	0.75	46	90	1	0.61	41	82	2
Zinc	0.66	38	80	1	0.47	40	74	4
Copper	0.69	43	82	0	0.59	45	82	0
Manganese	0.61	45	80	1	0.68	50	84	1
Iodine	0.44	40	71	2	0.42	32	68	3
Selenium	0.59	37	78	1	0.36	33	64	3
Chromium	0.50	31	72	2	0.47	28	68	3
Molybdenum	0.68	41	83	0	0.57	40	77	2
Retinol	0.41	47	73	3	0.45	41	70	4
α-carotene	0.53	43	71	1	0.66	39	80	0
β-carotene	0.55	40	76	0	0.64	37	78	1
Cryptoxanthin	0.46	30	69	1	0.53	32	78	1
Beta carotene equivalents	0.55	41	76	0	0.61	42	74	0
Retinol equivalents	0.50	41	77	4	0.60	41	78	2
Vitamin D	0.57	33	72	1	0.53	36	76	2
α-tocopherol	0.57	44	74	2	0.52	35	71	2
β-tocopherol	0.51	35	72	3	0.45	37	68	4
γ-tocopherol	0.37	27	65	3	0.50	36	71	2
δ-tocopherol	0.49	41	73	1	0.56	45	76	1
Vitamin K	0.60	40	79	1	0.56	39	75	2
Vitamin B_1_	0.56	37	72	1	0.57	38	73	1
Vitamin B_2_	0.66	38	85	1	0.41	35	71	4
Niacin	0.65	43	78	0	0.64	42	80	2
Vitamin B_6_	0.65	43	77	0	0.69	39	81	1
Vitamin B_12_	0.63	41	82	1	0.56	35	76	2
Folate	0.74	43	85	0	0.59	37	76	0
Pantothenic acid	0.60	40	79	1	0.44	34	70	2
Biotin	0.66	43	74	0	0.61	37	76	1
Vitamin C	0.71	37	86	1	0.60	32	69	0
Daidzein	0.52	38	80	3	0.65	43	77	0
Genistein	0.52	39	79	1	0.64	47	79	0
Ethanol	0.89	64	97	0	0.85	61	93	0
Median		39	77	1		37	75	2

a Spearman's rank correlation coefficients and the *p* values < 0.001 for energy and all nutrients.

b CCs and cross-classification for energy intake and total fat in %energy were calculated by using crude values; Percentages were based on the number of participants classified into the same, adjacent, and extreme categories by cross classification according to both quintiles using the web- and print-FFQ.

**Table 8:** Comparison of the web-FFQ and print-FFQ for energy-adjusted intake of food groups, based on correlation coefficient and cross-classification by quintile (%).

	Men (*n* = 98)				Women (*n* = 139)			
	CCs^a^	Same category	Same and adjacent category	Extreme category	CCs^a^	Same category	Same and adjacent category	Extreme category
Cereals	0.53	32	76	1	0.50	40	75	2
Rice	0.59	40	78	1	0.67	50	85	2
Potatoes and starches	0.52	36	73	0	0.45	38	71	2
Sugar	0.62	50	81	2	0.54	45	80	3
Pulses	0.47	42	74	3	0.64	46	78	1
Vegetables	0.56	36	72	1	0.56	32	73	1
Green and yellow vegetables	0.54	38	73	1	0.68	38	82	1
White vegetables	0.48	32	68	2	0.47	35	71	1
Pickled vegetables	0.68	42	83	1	0.61	35	78	1
Cruciferous vegetables	0.59	32	78	1	0.46	33	68	1
Fruits	0.44	32	67	1	0.61	35	82	1
Citrus fruit	0.49	37	73	1	0.47	29	72	2
Other fruit	0.48	31	70	1	0.65	46	81	1
Fungi	0.52	36	73	2	0.53	35	73	1
Algae	0.60	41	80	0	0.50	32	73	2
Fish and shellfish	0.59	35	74	0	0.49	37	76	4
Meats	0.54	32	73	0	0.48	32	71	1
Processed meat	0.63	39	81	1	0.70	44	83	1
Red meat	0.60	35	73	1	0.35	27	60	3
Poultry	0.54	28	73	2	0.37	27	68	4
Eggs	0.65	39	77	0	0.54	44	76	3
Milk and dairy products	0.66	40	80	1	0.48	37	69	1
Fats and oils	0.39	34	66	1	0.38	30	65	3
Confectionaries	0.59	41	82	2	0.55	36	72	1
Alcoholic beverages	0.81	53	91	0	0.65	44	74	0
Non-alcoholic beverages	0.60	39	74	1	0.64	41	81	1
Green tea	0.71	48	88	2	0.77	60	88	1
Coffee	0.77	51	88	0	0.79	55	91	0
Seasonings and spices	0.64	49	81	1	0.69	42	81	1
Median		38	74	1		37	75	1

a Spearman's rank correlation coefficients and the *p* values < 0.001 for all food groups; Percentages were based on the number of participants classified into the same, adjacent, and extreme categories by cross classification according to both quintiles using the web- and print-FFQ.

Finally, we conducted an additional stratified analysis of correlation coefficients between CCs of nutrient intake based on the 12d-WFR and each of the two FFQs by response location to the web-FFQ. CCs for energy and nutrients between the web- or print-FFQs and 12d-WFR were closely similar regardless of response location, with corresponding median CCs of 0.48 and 0.49, respectively, for men and 0.45 and 0.46, respectively, for women among private/residential respondents; corresponding values among onsite respondents were 0.48 and 0.46 for men and 0.38 and 0.40 for women (data not shown). Pearson's correlation coefficient between these CCs for nutrient intake based on the two FFQs and 12d-WFR were 0.7 and 0.8 for both men and women, respectively, among private/residential respondents, and 0.6 and 0.7 for men and women, respectively, among onsite respondents.

## Discussion

We examined the usability and validity of a web-FFQ developed as an online version of a print-FFQ used in the baseline survey of the JPHC-NEXT study. The accuracy of estimates obtained with the web-FFQ were comparable to those obtained with the print-FFQ.

Response times for the printed and web-based questionnaires, including overall information on lifestyle, were approximately the same for men, while the web questionnaire took slightly longer for women. The printed questionnaire is likely to require additional time to construct analyzable data over and above that allotted in this study, because of the need for staff review and follow-up for missing information or logical errors, as well as in the conversion of responses to electronic data. Considering conversion of data to electronic form, therefore, the web questionnaire was not inferior from the perspective of study efficiency. On the other hand, individuals who completed the web questionnaire on site because they did not have private or residential Internet access took longer to respond than individuals responding with their own Internet access. These individuals might have been unfamiliar with computer use, and this might have impacted their response time. However, because this was also true of response time with the printed questionnaire, the difference could not be explained by the interface alone. Rather, it might have also been because the onsite respondent was approximately 7 years older on average than those using their own Internet access. Moreover, many subjects said it was as easy or easier to respond using the web than the printed questionnaire. These results indicate that, with regard to study efficiency, the use of web-FFQs in cohort studies is reasonable, including use on site.

Correlations between the intake estimates obtained with the web-FFQ and 12d-WFR were moderate or better for many nutrients compared with previous validation studies of traditional printed FFQs among Japanese populations: these had median CCs ranging from 0.31 to 0.56 for target nutrients^33^ versus a median correlation for nutrients in our present study of approximately 0.5. These results are similar to previous results for the validity of web-FFQs compared with food records: the mean correlation coefficient across nutrients was 0.55 in a Canadian study of 69 men and women,^3^ 0.43 in an American study of 213 men and women,^4^ and 0.47 in a British study of 15 men and 34 women.^13^ The subjects in all of these studies were highly educated. Unlike any previous study of the validity of web-FFQs,^3^, ^4^, ^5^, ^6^, ^7^, ^8^, ^9^, ^10^, ^11^, ^12^, ^13^, ^14^, ^15^, ^16^ our present subjects were middle-aged and elderly individuals from the local population of the geographic areas covered by a cohort study, albeit that their participation was voluntary. Moreover, the number of days the reference method was used and the number of subjects were greater in our study than in these previous studies. The relatively much lower CC for estimated energy intake based on web-FFQ among women as well as print-FFQ might be caused by the food list on FFQ. As described in detail in our previous paper for validity of print FFQ,^18^ errors in estimates from the predetermined list were likely caused by the small contribution of individual foods to total energy intake. Our results show that the web-FFQ provided reasonable ranking for many nutrients and food groups in a range of intakes, as evidenced from the quintile cross-classification, albeit that agreement in estimating absolute intake was poor.

The characteristics of CCs for each nutrient and food group with the web-FFQ compared with the 12d-WFR were closely similar to those for the print-FFQ among both men and women, both when stratified by response location and combined. This finding indicates that the web- and print-FFQs provide similar levels of estimation accuracy for the same nutrients and food groups, and that intakes can be estimated in a similar fashion regardless of questionnaire format, whether by subjects with Internet access responding to a web-FFQ or subjects without Internet access responding to a print-FFQ.

In addition, a high proportion of rankings of intake estimates obtained with the web- and print-FFQs by quintile were classified into the same and adjacent quintiles for many nutrients and food groups (range for nutrients: 57–97% for men and 64–93% for women). A previous study that ranked nutrient intake estimates obtained with web- and print-FFQs by quartiles reported that 77–97% were classified into the same or adjacent categories.^7^ Our results compare favorably, even though these previous subjects were younger computer-literate individuals with relatively high education.^7^ Moreover, a previous study of the degree of concordance between nutrient rankings with two identical web-FFQs at 4-week intervals by quartiles (among 31 men and 69 women) reported that 87–98% were classified into the same and adjacent categories.^13^ By comparison, a study of concordance of nutrient rankings with two identical print-FFQs administered within the same year by quintiles (among 66 men and women) reported a range of 52–83%.^34^

Our study had several limitations. First, the time required to complete the printed questionnaire was self-reported. Moreover, because the web questionnaire could be completed in several separate sessions, response time included time for breaks and interruptions, although subjects taking longer than 24 h were excluded from the usability analysis. The actual web questionnaire response time may have been shorter, and the difference in total response time between the questionnaires may be overestimated. It is possible that the heightened degree of motivation and interest required of the participants of a validation study^1^ in their provision of complete and accurate information for this reference method might have had some effect of overestimating usability regarding time for completion. However, if present, the impact of this effect might be same for both the web- and print-FFQs. Second, because the mean interval between administration of the two different FFQs was 1.7 months (maximum, 4 months), the possibility that seasonal dietary changes affected the responses cannot be excluded.^35^ A previous comparison of web- and print-FFQs administered within 1 month showed a high level of concordance between rankings, although that study compared quartiles.^7^ This suggests that concordance may have been higher if the timing of administration were closer. Although cooking water could not be considered in these FFQs (in contrast to drinks, water, water content of food, noodle soup, and miso soup, which were included), this study also showed moderate validity for water content in men and women.

In conclusion, correlations between the intake estimates obtained with the web-FFQ and 12d-WFR indicated moderate validity for many nutrients and food groups in ranking of individuals by these intakes. These validities were closely similar to those of the print-FFQ, irrespective of the location of Internet access, with good concordance between individual rankings obtained with the two FFQs. These results suggest that the web- or print-FFQ can be used in epidemiological studies consistent with the location of the individual subject.

## Conflicts of interest

None declared.
